# A comparison of cognitive behavioral therapy for insomnia to standard of care in an outpatient substance use disorder clinic embedded within a therapeutic community: a RE-AIM framework evaluation

**DOI:** 10.1186/s13063-022-06885-7

**Published:** 2022-11-28

**Authors:** Traci J. Speed, Lisa Hanks, Gavin Turner, Evelyn Gurule, Alexandra Kearson, Luis Buenaver, Michael T. Smith, Denis Antoine

**Affiliations:** 1 Department of Psychiatry and Behavioral Sciences, Johns Hopkins University School of Medicine, 5510 Nathan Shock Drive, Suite 100, Baltimore, MD 21224 USA; 2 Perelman School of Medicine at the University of Pennsylvania, Philadelphia, USA

**Keywords:** Insomnia, Substance use disorder, Cognitive-behavioral therapy, Residential treatment, Recovery

## Abstract

**Background:**

Rates of substance use disorders (SUDs) continue to rise in the USA with parallel rises in admissions to outpatient SUD treatment programs. Insomnia symptoms reduce treatment adherence, trigger relapse, and generally undermine SUD recovery efforts. Cognitive-behavioral therapy for insomnia (CBT-I) is the first-line treatment recommended for chronic insomnia. No study has examined the effectiveness of CBT-I for individuals who recently entered an outpatient SUD treatment program embedded within a therapeutic community (i.e., long-term drug-free residential setting).

**Methods:**

A randomized controlled trial conducted at a SUD program embedded in a therapeutic community aimed to compare group-based CBT-I (gCBT-I) (*N* = 10) with the standard of care (SOC) (*N* = 11) among individuals who have SUDs and comorbid insomnia. We present a RE-AIM (reach, effectiveness, adoption, implementation, and maintenance) framework evaluation to provide empirical data on gCBT-I feasibility and facilitators and barriers of conducting an insomnia-focused clinical effectiveness study within a therapeutic community.

**Results:**

Participants in both study arms reported moderately severe insomnia symptoms at admission and reductions in insomnia symptoms over time. Among participants who completed the Insomnia Severity Index (ISI) beyond admission, ISI decreased to ≤ 8 (the clinical cutoff for mild insomnia) in 80% of individuals in the gCBT-I group compared with 25% of individuals in the SOC group. A RE-AIM framework evaluation showed initial success with Reach and Adoption while Implementation, and Maintenance were limited. Effectiveness was inconclusive because of challenges with recruitment, intervention integrity, and missing data that precluded meeting the planned recruitment and study aims and led to study termination. Coordination and communication with staff and leadership facilitated gCBT-I implementation, yet well-known CBT-I barriers including time- and resource-intensive sleep medicine training for interventionalists and maintenance of treatment integrity during an 8-week intervention limited gCBT-I sustainability.

**Conclusions:**

This analysis supports the feasibility of conducting behavioral sleep medicine research in outpatient SUD treatment programs embedded within therapeutic communities. Implementation of an insomnia-focused intervention was widely accepted by patients and providers and has potential to address insomnia symptoms in early SUD recovery. Addressing patient- and organizational-level implementation barriers may enhance the sustainability and scalability of sleep interventions and provide new hope to effectively treat insomnia among people living with SUDs.

**Trial registration:**

Clinicaltrials.gov: NCT03208855. Registered July 6, 2017https://clinicaltrials.gov/ct2/show/NCT03208855?term=NCT03208855&draw=2&rank=1

Contributions to the literature

- The RE-AIM implementation framework helped identify cognitive-behavioral therapy for insomnia implementation facilitators and barriers that are unique to residential substance use disorder (SUD) treatment settings.
- An insomnia-focused intervention was widely accepted by both patients and providers and has the potential to address insomnia symptoms in early SUD recovery.
- Addressing patient- and organizational-level barriers may enhance the sustainability and scalability of interventions targeting insomnia, a robust and potent risk factor for relapse, among people living with SUDs.

## Background

Rates of substance use disorders (SUDs) continue to escalate in the USA. Nearly 2 million individuals with SUDs seek treatment annually [1]; yet, 40–60% of these individuals will relapse within 1 year [2]. Upwards of 70% of individuals seeking SUD treatment report insomnia symptoms [3, 4], compared with 30% of the general population [5, 6]. Insomnia (i.e., difficulty falling or maintaining sleep, or non-restorative sleep despite adequate opportunity) may predate the onset of SUD [7–12], may arise from direct effects of drug use [13] or drug withdrawal, [14] and may persist for months to years after abstinence [13, 15–20]. Insomnia symptoms reduce treatment adherence, trigger relapse, and generally undermine SUD recovery efforts [21, 22]. Indeed, numerous studies indicate that subjective and objective measures of insomnia are among the best predictors of relapse regardless of the type of substance used [15, 20, 23–31]. While use of sleep aids is associated with greater treatment retention [32], their use in SUD treatment is often inappropriate [33] given their high abuse liability, and risk of falls, cognitive impairment, polypharmacy interactions, and rebound insomnia. Thus, non-pharmacological insomnia interventions provide a safer alternative in SUD treatment.

Robust evidence demonstrates that cognitive behavioral therapy for insomnia (CBT-I) is efficacious and safe across myriad psychiatric and medical disorders [34–40]. CBT-I combines stimulus control theory, sleep restriction therapy, sleep hygiene, and cognitive therapies [41] to alter maladaptive compensatory behavioral strategies (e.g., using alcohol or benzodiazepines) and cognitive processes that maintain disordered sleep. Expert consensus recommends CBT-I as the first line treatment for chronic insomnia [42–44]. The few studies that have evaluated CBT-I effectiveness during early SUD recovery have only studied individuals with one SUD, yet substance co-use is common among patients in SUD treatment [45]. The current evidence demonstrates post-CBT-I improvements in sleep quality and subjective insomnia severity among individuals in outpatient SUD treatment for alcohol use disorder (AUD) or on methadone treatment for opioid use disorder (OUD) [46–49]. To our knowledge, no study has evaluated the integration of CBT-I in an outpatient SUD program embedded within a therapeutic community (i.e., a long-term drug-free residential setting that promotes behavioral change and social reintegration) [50–52]. The therapeutic community model has demonstrated significant and sustained improvements in SUD and psychosocial outcomes; notably longer treatment duration correlates with improved outcomes [53]. Addressing insomnia for individuals in therapeutic communities is important because insomnia is a common, yet modifiable risk factor of substance relapse. Also, promoting good sleep aligns with the therapeutic community treatment model to assimilate social norms and instill self-reliance [54]. The effectiveness of insomnia interventions within therapeutic communities remains unknown.

To address this crucial gap, we conducted a randomized controlled trial (RCT) evaluating the effectiveness of group-based CBT-I (gCBT-I) compared with the standard of care (SOC) among individuals with any primary SUD and comorbid insomnia at an outpatient SUD program embedded within a therapeutic community. We implemented a group-based intervention as commonly used in intensive outpatient, inpatient, stepped-care, and residential SUD recovery programs because group treatment is more cost- and time-efficient and promotes social reinforcement of target behaviors [55, 56]; gCBT-I is equally efficacious as individual treatment [57].

Following early termination of the RCT for reasons discussed below we conducted an implementation science evaluation of the gCBT-I intervention. Implementation science framework evaluation can help examine facilitators and barriers of real-world implementation and sustainability and standardize reporting outcomes [58, 59]. Reach, Effectiveness, Adoption, Implementation, and Maintenance (RE-AIM) is a well-established framework used effectively in substance use services research [60–62]. While the application of implementation science frameworks to evaluate health behavior interventions is common [58, 61, 63], their use to evaluate the dissemination of sleep interventions in clinical settings is limited [64]. We present an implementation science evaluation utilizing the RE-AIM framework to provide empirical data on gCBT-I feasibility and to evaluate facilitators and barriers of gCBT-I implementation to promote dissemination and sustainability of sleep interventions as a component of early SUD recovery.

## Methods

### Setting

The Helping Up Mission (HUM) is a faith-based residential therapeutic community [52, 54, 65] established in 1885 that provides holistic and comprehensive treatment services with a focus on blending spirituality with medicine. HUM provides housing, access to health care (i.e., medical, mental health, and dental), and rehabilitative services to men from the greater Baltimore area. There is a steady state census of over 525 men residing at HUM; residents can live there for 1 year while in HUM’s Spiritual Recovery Program. HUM established a pilot women’s program in late 2019 and expanded its women’s services in 2021. Throughout the program, residents participate in the faith-based classes and contribute to different aspects of HUM’s daily operations (i.e., cook, janitor, security) and in the latter portion of the first year, residents can engage in a job training program and obtain internships. Given that HUM residents have unstable housing, low socio-economic backgrounds, and severe SUDs, housing for the entire year provides an opportunity for restoration beyond only SUD treatment. HUM’s proximity to The Johns Hopkins Health System has helped foster a community-academic partnership.

The Cornerstone Clinic (CC) is a Johns Hopkins University School of Medicine operated state-licensed behavioral health program accredited by the Commission on the Accreditation of Rehabilitation Facilities (CARF) that provides on-site SUD treatment to residents of HUM since 2012. CC is not a level III facility for substance withdrawal (residents who require medical management for acute withdrawal are transferred to the nearest emergency department) and at the time of this study provision of medications for OUD (mOUD) was unavailable due to standard HUM therapeutic community principles. CC provides conventional outpatient behavioral health care where the intensity of treatment is based on American Society of Addiction Medicine (ASAM) criteria [66]. Aligning with standard HUM therapeutic community principles, CC clients are not able to take benzodiazepine or benzodiazepine-like sleep aids and certain psychotropic medications are restricted due to their abuse liability (e.g., quetiapine). CC’s practice guidelines are grounded within an evidenced-based model of reinforcement-based treatment (RBT), which employs a client-centered approach to treatment. RBT begins with a comprehensive functional analysis of drug use behavior and is accompanied by goal setting and positive reinforcement for treatment engagement. The RBT modality has been studied across different SUD treatment populations with good efficacy [67, 68]. HUM has recognized CC as an essential component of the treatment plan for its residents. All HUM clients are eligible for CC, and they are prioritized by key markers that are indicative of higher SUD acuity (i.e., HIV status, intravenous drug use, recent discharge from a rehabilitation program, hospital, or penal facility). Most clients (~98%) admitted to CC meet criteria for intensive outpatient treatment (IOP) (ASAM II.1). These clients report to CC for a total of at least 9 hours of group and individual SUD treatment dispersed over 4 days per week. Within approximately 12 weeks once ASAM criteria for IOP level are no longer applicable, clients step down to ASAM level 1 outpatient treatment, which consists of 3 hours total per week of group and individual treatment. On average, clients receive IOP and outpatient treatment for a total of 4 months. Clients who are administratively discharged from HUM (i.e., if they relapse) are simultaneously discharged from CC.

CC clients provide randomized urine samples for toxicology testing in conjunction with their standard clinic treatment. At the time of this report, clients submitted weekly randomized tests, but the clinic has since adopted a smart testing system recommended by the American Society of Addiction Medicine [69]. Urine samples are collected on-site and sent to an outside laboratory for testing. Results are returned through the patient’s electronic medical record which only CC staff reviews. The following substances are part of the standard qualitative panel for the clinic: opioids (including fentanyl), methadone, marijuana, alcohol (ETs and ETg), cocaine, amphetamines, and benzodiazepines (300 ng/ML). LCMS confirmatory testing are available as needed. Results remain confidential among CC providers in accordance with the Code of Federal Regulations 42, part 2 (42CFR).

To address client scheduling and attendance problems, CC began compiling weekly statistics on attendance and intake data that were distributed to HUM staff as a foundation for a collaborative, weekly face-to-face staff meeting. This ongoing academic-community partnership has yielded operational strategies that improved the mean IOP attendance rate from 49% in FY14 to 80% in FY18. This attendance rate has allowed for enhancement and broadening of services (e.g., gCBT-I) to address critical gaps in treatment. One such gap was highlighted when preliminary data revealed that 34% of HUM clients met criteria for clinical insomnia at intake. Given the potential for insomnia to trigger relapse, this trial aimed to evaluate the effectiveness of embedding this efficacious insomnia-focused behavioral health intervention into the program.

### Participants

The study was conducted by CC staff at HUM. CC has an average census of 50-60 clients, or approximately 33% of the HUM residents in the Spiritual Recovery Program. Participants were enrolled between August 28, 2017, and October 5, 2019. Treatments and follow-up were completed by April 13, 2020. HUM residents aged 18–65 years old are referred to CC within 2 weeks of HUM admission. CC clients who signed a HIPAA-compliant permission form to be screened for research studies complete patient-reported outcome measures (PROMs) within 1 week of CC admission.

CC clients were eligible for the RCT based on the following inclusion criteria: (1) enrolled in CC for less than 3 weeks; (2) resident at HUM; (3) has a SUD; (4) ≥ 18 years old; (5) Insomnia Severity Index (ISI) ≥ 8 at CC admission; (6) self-report of sleep onset latency (SOL) > 30 min, wake after sleep onset (WASO) > 30 min, or a combination of SOL and WASO > 30 min; and (7) self-report insomnia symptom frequency > 3 nights/week for > 1 month. Exclusion criteria included the following: (1) history of bipolar disorder; (2) history of seizure disorder; (3) suicidal ideation; (4) acute alcohol withdrawal requiring medical attention; (5) medical, psychiatric, or concomitant condition interfering with treatment or requiring hospitalization; and (6) inability to provide informed consent.

### Data collection and outcome measures

#### Sleep measures

The Structured Interview for Sleep Disorders (SIS-D) [70] was adapted to DSM-5 criteria and implemented to diagnose and classify insomnia and identify potential intrinsic sleep disorders, including sleep apnea. Subjects who screened positive for sleep apnea were informed and referred to a primary care physician for clinical evaluation. If they did not have a primary care physician, CC staff helped establish care. Individuals who screened positive for apnea were eligible to participate in the study to mirror real world clinical settings.

#### Insomnia

The Insomnia Severity Index (ISI) is a validated insomnia screening instrument with good psychometric properties [71] previously used in studies of SUD [72–74]. The ISI has well established cut-off criteria demarcating clinical insomnia [75, 76]; a total score of 8–14 suggests subthreshold insomnia, 15–21 suggests moderately severe clinical insomnia, and 22–28 suggests severe clinical insomnia.

#### Sleep diary

Diary measures of sleep have become standard methods for assessing outcomes in insomnia clinical trial literature [77, 78]. Prospective daily monitoring has been shown to minimize memory biases that threaten the validity of global retrospective ratings. Diary information was collected with paper diaries which are widely used in sleep/insomnia research and have demonstrated sound psychometric properties [78, 79]. While studies in individuals with primary insomnia have been shown to overestimate SOL and WASO, diaries provide a valid, relative index of sleep continuity disturbance that is sensitive to change because overestimation remains consistent over time [34, 80–82]. Comparable to most outpatient and/or residential SUD treatment programs, HUM residents are beholden to a black out period in which they do not have access to personal phones or computers for the first 45 days of treatment. Use of paper diaries allowed participants to maintain daily sleep diaries independent of access to mobile technology. Diary questions included bedtime, time of final awakening, time out of bed, estimated SOL, WASO, and visual analog scale (VAS) ratings of subjective sleep quality and fatigue. The subjective measures of sleep (e.g., SOL, WASO, sleep efficiency [SE], and total sleep time [TST]) were averaged across a one-week monitoring period to provide a weekly estimate used for statistical computations.

#### Alcohol and drug use

Clients are diagnosed with primary and secondary SUDs based on clinical assessment using DSM-5 criteria [83].

#### RE-AIM outcome measures

While we initially intended to enroll 80 participants and have 40 completers, we identified challenges that led us to end the study early and conduct an implementation framework analysis. The RE-AIM framework (Table 1) guides the presentation of our results.

**Table 1:** RE-AIM outcome measures

	I	P	O	
**Reach**	x			% eligible CC clients who consented to study
			x	% enrolled participants who attended 1st gCBT-I session
**Effectiveness**	x			ISI at post-intervention and 3-month follow-up
	x			Subjective sleep parameters at post-intervention
**Adoption**		x		% eligible providers who led gCBT-I
**Implementation**	x			Adherence to sleep diary completion
	x			% gCBT-I participants who attended 8 sessions
**Maintenance**	x	x	x	gCBT-I implementation facilitators and barriers

*I* individual, *P* provider, *O* organizational, *CC* Cornerstone Clinic, *gCBT-I* group cognitive behavioral therapy for insomnia, *ISI* Insomnia Severity Index

Reach was measured by two metrics. At the individual level, we measured the percent of CC clients who consented to the study with the numerator defined as the number of eligible CC clients who consented to the RCT study. The denominator included eligible CC clients who were scheduled for RCT screening visit based on a CC admission ISI score ≥ 8 and eligible medical history and declined participation. At the organizational level, we measured the percent of enrolled participants who attended gCBT-I session 1. We considered this an organizational outcome measure given the nuances of care coordination involved in scheduling participants for the gCBT-I session.

Effectiveness was measured as our a priori RCT primary outcomes: ISI and subjective sleep parameters (i.e., SOL, WASO, SE, TST) at post-intervention.

Adoption was measured at the provider level. The percent of eligible providers who conducted gCBT-I was calculated as the number of CC providers who completed behavioral sleep medicine training to become gCBT-I interventionists.

Implementation has two metrics. Adherence to sleep diary completion was defined as the percent of participants who completed weekly sleep diaries during the 8-week gCBT-I intervention or 8-week SOC period, and gCBT-I completion was defined as the percent of those enrolled in the intervention arm who attended all 8 gCBT-I sessions.

Maintenance is reported as the implementation facilitators and barriers for sustainability and scalability.

### Study procedures

#### Screening

The Johns Hopkins Institutional Review Board approved the study protocol. All participants provided written informed consent. CC clients who had ISI ≥ 8 on admission were approached by research staff to determine their interest in the research study. CC clients who expressed interest were scheduled for one screening visit to establish eligibility for participation. Screening occurred outside of weekly scheduled CC individual and group therapy, so it did not interfere with IOP treatment and included research diagnostic interviews with an adapted SIS-D [70]. Participants completed 5 multiple-choice questions about the study to assess their understanding of the information provided prior to enrollment.

#### Randomization

The allocation sequence was generated using Research Randomizer [84], which provides a 1:1 computer-generated allocation of random numbers. No factors were used for stratification or planned blocking. The allocation sequence was maintained on a password protected, institution-encrypted, HIPAA-compliant cloud-based server. After participants provided their consent, research staff randomized participants to gCBT-I or SOC based on the next available arm on the sequentially numbered list.

#### Interventionists

SOC was provided by CC behavioral health providers including psychiatrists or licensed masters-level mental health counselors (e.g., LCPC) who have experience in general behavioral medicine interventions and substance use. Two interventionists with specialized sleep medicine training led each gCBT-I session. gCBT-I interventionalists were either CC counselors (1 woman, 1 man), Johns Hopkins psychiatrists (2 women, 1 man), or an advanced psychology doctoral candidate (1 woman). Interventionists received training in behavioral sleep medicine through a 3-day CBT-I workshop (University of Pennsylvania) and/or an extensive 3-month training period that included formal didactic lectures on sleep, assigned readings on sleep medicine, hands-on training, review of treatment manuals, and observations of CBT-I. A CC provider was present for each gCBT-I session.

### Treatment protocols

#### gCBT-I

We administered three separate closed-group (4–5 participants/group) standardized 8-session (60 min/session) multicomponent interventions based on our published treatment manual [85] adapted for group-based treatment. Groups were conducted between November 1, 2017–January 4, 2018, March 7, 2018–April 25, 2018, and October 9, 2019–November 27, 2019. Major intervention components included sleep restriction therapy, stimulus control therapy, cognitive therapy for insomnia, and sleep hygiene education. Sessions included reviewing each individual’s paper diary data, charting progress, setting measurable goals, discussing adherence, and reinforcing learned skills. Each gCBT-I session counted towards one weekly group therapy session.

#### SOC

As previously mentioned SOC included 9 total hours of weekly services comprised of group and/or individual behavioral health sessions. Participants randomized to SOC were instructed how to complete daily sleep diaries and where to handoff diaries each week and receive a new paper diary.

### Statistical analysis

Baseline participant sociodemographic and clinical characteristics were summarized and stratified by gCBT-I and SOC groups. Continuous variables were summarized as mean and SD and analyzed using a *t*-test for approximately normally distributed variables. Categorical variables were summarized as number (%) and analyzed using a *χ*^2^ test. Statistical significance was assessed at the 0.05 level, and all tests were two-sided. All analyses were conducted using SPSS (IBM statistics, Version 24).

## Results

### Reach

**Fig. 1:**
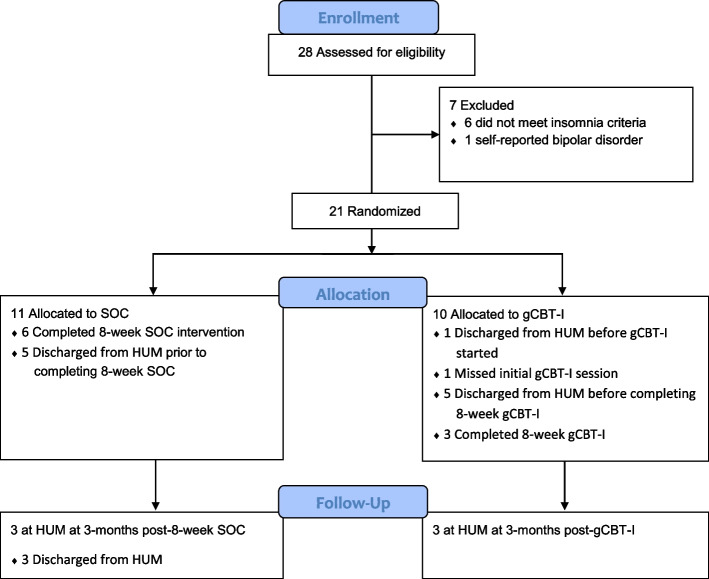
CONSORT flow diagram

Of the 29 participants who attended the screening session, 28 (96.5%) provided informed consent, and of those, 21 (75%) were eligible for enrollment. The consort diagram (Fig. 1) shows participant enrollment and allocation. Table 2 shows the baseline demographic and clinical characteristics of the randomized participants. There were no statistically significant differences between groups including baseline ISI. Of the participants enrolled in the gCBT-I treatment arm, 8 (80%) attended the first gCBT-I session. One participant did not attend the first gCBT-I session and was ineligible to continue in the study. One participant was discharged from HUM prior to his first gCBT-I session.

**Table 2:** Demographic and clinical factors

	Total	SOC	gCBT-I	*p*-value
	*N* = 21	*N* = 11	*N* = 10	
Age in years (SD)	40.6 (13.2)	45.6 (14.1)	35.1 (10.0)	.07
Gender (%)				
Male	21 (100)	11 (100)	10 (100)	NA
Race (%)				.29
American Indian	1 (4.8)	0 (0)	1 (10)	
> One race	1 (4.8)	0 (0)	1 (10)	
Black/African American	8 (38.1)	5 (45.5)	3 (30)	
White/Caucasian	11 (52.4)	6 (54.5)	5 (50)	
Ethnicity (%)				
Not Hispanic or Latino	0 (0)	0 (0)	0 (0)	NA
Employment in past 90 days (%)				.14
Full-, part-time, or student	9 (42.9)	6 (54.6)	3 (30)	
Retired	1 (4.8)	1 (9.1)	0 (0)	
Unemployed/disability	11 (52.4)	4 (36.4)	7 (70)	
Income (%)				.36
$0–$15,000	11 (52.4)	4 (36.4)	7 (70)	
$15,001–$30,000	2 (9.5)	2 (18.2)	0 (0)	
$30,001–$45,000	2 (9.5)	1 (9.1)	1 (10)	
$>45,000	1 (4.8)	1 (9.1)	0 (0)	
Missing	5 (23.8)	3 (27.3)	2 (20)	
Relationship status (%)				.12
Never married	13 (61.9)	6 (54.5)	7 (70)	
Married	2 (9.5)	0 (0)	2 (20)	
Divorced/separated	5 (23.8)	4 (26.4)	1 (10)	
Widowed	1 (4.8)	1 (9.1)	0 (0)	
Smoking tobacco				
No	2 (9.5)	2 (18.2)	0 (0)	
Yes	19 (90.5)	9 (81.8)	10 (100)	.17
Number cigarettes smoked (%)				
0	2 (9.5)	2 (18.2)	0 (0)	.08
1–10	11 (52.4)	3 (27.3)	8 (80)	
11–20	7 (33.3)	5 (45.5)	2 (20)	
> 20	1 (4.8)	1 (9.1)	0 (0)	
Primary SUD				.19
Alcohol	4 (19)	2 (18.2)	2 (20)	
Opiates	14 (66.7)	6 (54.5)	8 (80)	
Marijuana	1 (4.8)	1 (9.1)	0 (0)	
Cocaine	2 (9.5)	2 (18.2)	0 (0)	
Days since last drug use (SD)	27.2 (5.1)	27.1 (5.9)	27.3 (4.3)	.99
Prior detox or rehab admissions (SD)	4.0 (3.0)	4.2 (3.6)	3.8 (2.4)	.73
ISI	17.0 (5.4)	17.3 (5.9)	16.7 (5.3)	.82

*SOC* standard of care, *gCBT-I* group cognitive behavioral therapy for insomnia, *SUD* substance use disorder, *ISI* Insomnia Severity Index, *p < 0.05

### Effectiveness

**Fig. 2:**
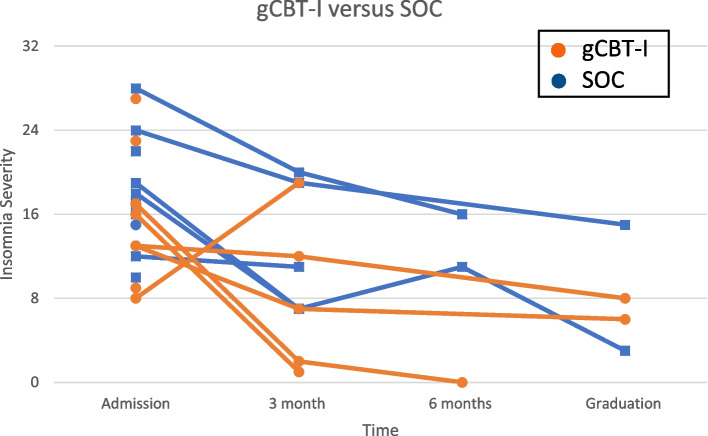
Insomnia Severity Index over time comparing gCBT-I versus SOC

**Fig. 3:**
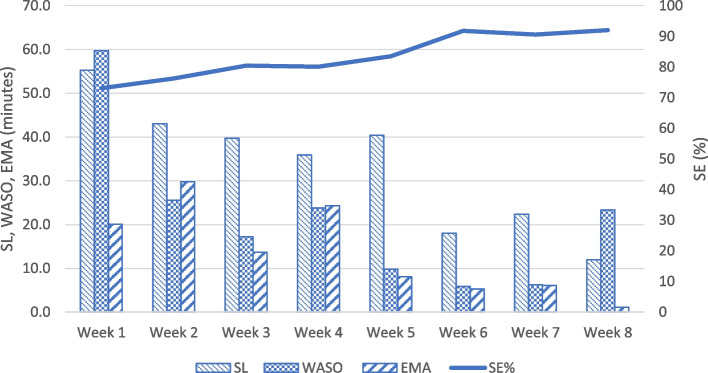
Pre-to-post-intervention diary-derived sleep parameters of gCBT-I participants

Study participants self-reported moderately severe insomnia symptoms at admission (Table 1). Figure 2 shows trends in ISI across participant’s treatment duration. Most participants reported reductions in insomnia symptoms while in CC treatment. Among participants with ISI scores beyond admission, insomnia severity decreased below or at the clinical cutoff for mild insomnia (ISI ≤ 8) in 4 out of 5 individuals in the gCBT-I group, compared with one out of 4 individuals in the SOC group. The participants who graduated from CC (3 gCBT-I and 2 SOC participants) remained in outpatient treatment for 171 ± 25 days. gCBT-I showed pre-post improvements in subjective sleep outcomes including SOL, WASO, and SE (Fig. 3). Additionally, TST improved from 5 h at pre-intervention to 6 h at post-gCBT-I; TIB appropriately decreased from 7 h at pre-intervention to 6.6 h at post-gCBT-I. Sleep diary data was not collected beyond gCBT-I sessions.

For this RE-AIM analysis, we limited effectiveness to post-intervention sleep outcomes, yet it is important to note that in planning the clinical effectiveness study in 2017, our a priori primary outcomes included sleep outcomes through 6-month post-intervention, and our a priori secondary outcomes included (1) attrition rates in SUD treatment at 3- and 6-month follow-up and (2) depressive symptoms, pain, and pain catastrophizing at 3- and 6-month follow-up. While we also intended to measure SUD relapse rates through 3-month follow-up (*unpublished trial protocol*), we did not report this outcome measure on clinicaltrials.gov. In 2019, after completing 2 rounds of unblinded gCBT-I interventions, we recognized gaps in our study design that limited gCBT-I treatment integrity (see Implementation), thus limiting inferences of our secondary outcomes. We subsequently removed depression and pain as outcomes on clinicaltrial.gov. SUD outcomes are not reported for similar reasons.

### Adoption

CC employed 4 behavioral health counselors across the study duration. One counselor completed behavioral sleep medicine training prior to study onset and one counselor had completed behavioral sleep medicine training prior to CC employment. The 2 other counselors did not complete behavioral sleep medicine training due to financial and time constraints.

### Implementation

Three participants (30%) randomized to gCBT-I completed all 8 sessions within 8–9 weeks (56–63 days). One participant (10%) was discharged before the intervention began. Six participants (60%) were discharged from HUM before completing the intervention; one of those six participants was discharged between weeks 7 and 8 of CBT-I. gCBT-I participants completed an average of 5 sessions and remained at HUM for an average of 37 days after the first gCBT-I session. Following the first gCBT-I session, 100% of gCBT-I attendants brought their completed paper sleep diaries to the sessions. None of the participants in the SOC group returned his sleep diaries.

### Maintenance

During weekly research meetings with CC mental health providers and monthly meetings with HUM leadership, we discussed strategies to improve study workflow. We identified gCBT-I implementation facilitators at the patient, provider, and organizational level (Table 3). At the patient level, (1) patients expressed interest in learning about and treating their insomnia as evidenced by nearly ubiquitous enrollment and adherence to CBT-I treatment recommendations. At the provider level, (1) behavioral health therapists, psychiatry residents, and advanced postdoctoral candidates demonstrated willingness to learn CBT-I and serve as interventionists. We consistently had two interventionists attend each gCBT-I session. At the organizational level, (1) we had a well-established clinical research infrastructure to identify eligible CC clients; (2) we planned and coordinated gCBT-I sessions to avoid scheduling conflicts with CC and HUM activities; and (3) we maintained consistent communication between HUM and CC leadership. Specifically, clients who provided informed consent upon entering CC completed a battery of PROMs assessing psychosocial factors within 1 week of CC admission. Based on an ISI ≥ 8, CC staff scheduled eligible clients for a screening/informed consent session in evenings or on weekends. Research staff sent the list of consented participants via a HIPAA-compliant correspondence to CC staff who scheduled the clients for gCBT-I. gCBT-I sessions were included on CC clients’ weekly schedules to promote treatment adherence. Similar to the screening appointment, gCBT-I sessions were scheduled at a pre-determined time that did not coincide with scheduled CC or HUM activities. Additionally, CC staff educated HUM staff on the potential clinical utility of CBT-I and reviewed initial challenges and successes with the intervention. Thus, HUM was amenable to modifying curfew rules and adapting space for participants to use before their designated (i.e., delayed) bedtimes. HUM communicated these adaptations with nighttime security so that participants had adequate opportunity to comply with the CBT-I protocol. No study harms or unintended effects were reported by participants from either group, staff, or leadership.

**Table 3:** Facilitators and barriers of conducting an insomnia intervention within a therapeutic community

Level	Facilitators	Barriers
**Patient**	Patient interest in learning about sleep and treating insomnia	Maintenance of treatment integrity because participants missed gCBT-I sessions
**Provider**	Staff and students willing to complete sleep medicine training and conduct gCBT-I sessions	Efforts to minimize workload for staff that limited opportunity to recruit gCBT-I participants
		Staff and volunteer turnover that delayed recruitment
		Sleep medicine training that was time- and resource-intensive
**Organizational**	Well-established clinical research infrastructure within the behavioral health program	Limited opportunities to collect weekly paper sleep diaries as part of standard clinical practice
	gCBT-I sessions that did not conflict with scheduled SUD treatment	Time gap from behavioral health program enrollment to 1st gCBT-I session
	Consistent communication with staff and leadership in the behavioral health program and therapeutic community	

*RCT* randomized controlled trial, *gCBT-I* group cognitive behavioral therapy for insomnia

In this context, we also identified gCBT-I implementation barriers. At the patient level, (1) we found that it was impractical to exclude participants for missing one session, despite our concerns with maintaining treatment integrity. Yet, it was also impractical to lead a group-based cognitive session if participants had not adequately learned sleep-focused behavioral therapy. At the provider level, (1) conducting screening/consent during non-workday hours limited recruitment opportunities; (2) the onboarding of new research staff and volunteers to conduct screens led to recruitment delays; and (3) sleep medicine training was time- and resource-intensive (e.g., 3-day seminar and 3-month training curriculum). Specifically, we mostly relied on research volunteers to conduct the consent/screening process in evenings and on weekends. Volunteer turnover led to frequent onboarding and delays in screening. The lack of volunteers to conduct screening during non-work hours was a primary reason for the 2- and 6-month-long gaps between the 3 gCBT-I treatments. While our interventionists had ample access to sleep medicine training, many behavioral health clinics are not associated with an academic medical center, thus limiting scalability. At the organizational level, (1) we did not implement a sufficient method to collect paper sleep diaries from the SOC group and (2) randomizing 4-5 participants to gCBT-I or SOC took several weeks and delayed treatment. Regarding paper diaries, we considered having CC therapists collect weekly sleep diaries, yet suspected this could interfere with the SOC treatment arm (i.e., therapists could inadvertently provide sleep education while discussing the paper diary with their clients). Remuneration proved an insufficient reward, but care was taken to not offer an amount that that could be viewed as coercive in relation to other contingency rewards available in the clinic at the time. Regarding the time gap from admission to intervention, it took an average of 25 ± 10 days from initial CC visit to enroll participants in the study; accumulating a cohort of 4–5 individuals for the gCBT-I intervention arm took an average of 51 ± 23 days from initial CC visit to the first gCBT-I session. Thus, CC clients started the intervention approximately half-way through an average of 101 ± 76 days of treatment and we were missing a critical window to intervene in acute SUD recovery. Additionally, days until gCBT-I completion (51 days to first gCBT-I session plus 57 days of the 8-week intervention) equaled days in CC treatment and precluded our ability to measure 3- and 6-month follow-up of sleep and SUD-related outcomes.

In summary, while we had sufficient recruitment pool to enroll 80 participants in a 2-year period, we only accrued 25% of our targeted sample in that period. Implementation barriers led to gaps in (1) recruitment; (2) data collection (e.g., missing sleep diaries from the SOC group, no long-term follow-up outcomes); and (3) intervention integrity (e.g., delayed time to gCBT-I, poor treatment adherence). We recognized that optimizing recruitment, gCBT-I treatment integrity, and data collection for the successful completion of an effectiveness trial were not possible without substantial changes to our protocol and additional funding to hire research coordinators. Hence, we terminated the study with the intent of using this implementation framework to inform future studies.

## Discussion

Insomnia is common in SUD and a robust predictor of relapse. CBT-I is recommended as first-line treatment for chronic insomnia in adults, yet it is not widely disseminated in real-world clinical settings, including behavioral health programs [86]. Our implementation science evaluation of a RCT that compared gCBT-I with SOC in an outpatient SUD program embedded within a therapeutic community showed initial success with *Reach* (96.5% of screened clients provided informed consent to participate in the research study; 80% attended the first gCBT-I session) and *Adoption* (50% of counselors completed sleep medicine training). *Effectiveness* was inconclusive. Due to our small sample size, missing data, and study termination inferences about effectiveness were not possible. We did not observe between group differences in insomnia severity at post-treatment. We observed pre-post improvements in the secondary outcomes of sleep parameters in the gCBT-I intervention arm, although we did not sufficiently collect comparator data. Poor treatment integrity precluded analysis of *a priori* secondary outcomes: attrition, SUD relapse rates. *Implementation* at the individual and organization level was also limited; only 30% of participants in the gCBT-I intervention completed the 8-week intervention. Regarding *Maintenance,* we faced well-known CBT-I implementation barriers, including limited time and resources for interventionists to complete sleep medicine training and a nearly 2-month delay from CC entry to gCBT-I onset, that limit sustainability and dissemination of an 8-session gCBT-I intervention in real-world residential therapeutic communities. Conversely, communication between HUM and CC was optimal for study implementation, which will aid future iterations of the study.

The RE-AIM framework addresses the external validity of clinical research as a method to facilitate translation of research into clinical practice [87, 88]. A significant chasm exists between the robust efficacy of CBT-I in clinical trials and its dissemination into clinical practice [86]. This work uniquely contributes to the literature by identifying common and unique CBT-I implementation facilitators and barriers in the context of an academic-community partnership and therapeutic community setting. Implementation facilitators included a pre-existing behavioral health research program, adequate synergy between the research program and the various community stakeholders (e.g., clients, staff, volunteers, providers, and leadership) to initiate an insomnia intervention, a standardized screening protocol within the behavioral health program to access the target population, and frequent communication between various stakeholders.

In addition to previously discussed common barriers, the RE-AIM framework shed light on critically important barriers specific to conducting an RCT within a behavioral health program. First, participants did not have sufficient access to the intervention despite their interest. One client was discharged from care before initiating gCBT-I and six clients were discharged from HUM before completing the intervention. Inadvertently, our strategy to use a group-based intervention delayed treatment onset because it took several weeks to randomize 4–5 participants per group. Additionally, we experienced gaps in recruitment due to volunteer turnover. Thus, future studies may need to consider alternative research designs to avoid delays in intervention onset and gaps in recruitment. Second, requiring attendance at every gCBT-I session increased treatment attrition. Recognizing that clients may miss a session due to conflicting medical or psychosocial appointments that are critical to their stabilization early in treatment, we modified this requirement to allow participants to miss up to 4 out of 8 sessions. An unresolved barrier remains, how to balance treatment integrity with leniency for missing groups. Third, participant remuneration for return of weekly paper sleep diaries did not suffice in the SOC group; hence, we had inadequate comparator data to evaluate our secondary outcomes. Finally, our study targeted CC clients who reported mild-to-severe insomnia at CC admission. We excluded HUM residents who did not attend CC and CC clients who may have experienced new onset insomnia beyond admission. Thus, we potentially missed a large cohort of residents in the therapeutic community program who could benefit from an insomnia intervention. Expanding the treatment to all HUM residents could address the previously mentioned barrier of insufficient study participants for a RCT yet would require more resources and an improved triaging system.

This study also quashed our initial postulates about the feasibility of protocol implementation. Specifically, we expected that an inhospitable sleeping environment (e.g., roommates snoring, lack of white noise, inability to regulate room temperature or light) within a residential setting would uniquely impede participants’ willingness and ability to adhere to the CBT-I protocol [42, 89]. Participants did not substantiate this concern suggesting that communal sleeping environments may not be a barrier to conducting further insomnia interventions within residential therapeutic communities. Evidence of pre-post improvements in sleep parameters provides additional support that CBT-I treatment adherence is potentially feasible in residential settings.

Given that chronic insomnia is a well-established predictor of SUD relapse, several RCTs have been conducted in outpatient settings among community-dwelling individuals and Veterans living with a SUD with the hope that CBT-I positively influences SUD recovery. To date, the extant literature has examined CBT-I in alcohol-dependent adults in early recovery [46–48], young adults who are actively drinking [90], and patients on methadone treatment for OUD [49]. Collectively, these studies found that CBT-I improved subjective insomnia symptoms relative to control but did not modify SUD outcomes. Similarly, we found that individuals who received the gCBT-I intervention reported pre-to-post improvements in subjective sleep including sleep continuity, sleep efficiency, and total sleep time. Contrary to the literature, we did not find between-groups differences in self-reported insomnia severity, and the present study did not analyze SUD outcomes. A consistently reported limitation across the extant literature is that studies have been underpowered to detect changes in substance use [46–49, 90]. We speculate that additional nuanced barriers may limit CBT-I’s effectiveness at reducing SUD severity. For instance, except for Arnedt and colleague’s [46] CBT-I adaption to include education about alcohol’s effects on sleep, to the best of our knowledge, none of the studies adapted CBT-I to focus on SUD; although participants may have received concurrent SUD treatment [48]. RE-AIM or other implementation science frameworks may be useful planning tools to guide iterative changes to CBT-I to more effectively address the relationship between insomnia and substance use among SUDs populations.

Our study is the first to evaluate CBT-I among individuals with multiple types of primary SUDs, and often co-occurring SUDs (CODs), and to offer CBT-I for individuals living in a residential therapeutic community. Sleep disturbances are highly prevalent among individuals in residential therapeutic communities. Wilkerson and colleagues recently showed that most clients who entered a community IOP for SUD treatment had significant sleep disturbances, regardless of primary SUD or COD, and that insomnia severity, but not sleep quality or sleepiness, predicted IOP treatment completion [45, 91]. This is important because it suggests that insomnia-focused interventions such as CBT-I may widely benefit individuals in intensive levels of SUD care regardless of type of primary SUD or CODs and buttresses support for further CBT-I implementation science studies within IOPs, residential facilities, and therapeutic communities.

Future directions to enhance CBT-I implementation and sustainability in real world SUD clinics may focus on expanding reach, identifying target populations, decreasing burdens of time and access to services, and incorporating the intervention into existing clinical workflow while reducing demand on clinical resources and staff. Using the RE-AIM framework, we have identified potential adaptations for future clinical studies at our current study site. These include (1) engaging clients and other stakeholders in intervention planning, (2) transitioning to a brief behavioral treatment for insomnia (BBTI) that would remove the barrier of specialty sleep medicine training and time constraints [92, 93], (3) incorporating a sleep assessment during the behavioral health intake as a billable service, (4) expanding eligibility criteria to include all therapeutic community residents regardless of insomnia scores at admission, (5) offering an introductory insomnia educational session early in SUD treatment to increase client interest in insomnia therapy, and (6) integrating the electronic health record with HIPAA and 42CFR-compliant web applications (i.e., REDCap) to more efficiently collect data on insomnia and substance use related outcomes. Additionally, mixed-methods analyses that collect and triangulate quantitative and qualitative data from clients and other stakeholders will be essential to inform future implementation and adaptations of insomnia interventions.

Our results must be considered in the context of study limitations which can be accounted for in future studies. First, it does not meet the CONSORT extension guidelines for randomized pilot and feasibility trials as feasibility was not an a priori primary outcome. Second, the limited number of participants accrued due to study termination precludes any relevant group comparisons; thus, the completed study diverged from the planned RCT regarding recruitment and study aims as inferences about intervention effectiveness regarding sleep and SUD outcomes were not possible. Third, the study was conducted over a 2-year period and only in men (HUM was a men’s therapeutic community through 2019). Although, there were no significant changes among infrastructure, clinical staff, and leadership at CC or HUM during the 2-year period, it is possible that internal or external variations across the long study duration impacted results between groups. Also, women could experience different outcomes. Fourth, to protect client confidentiality and to maintain clients’ satisfaction levels, we did not audio-record gCBT-I sessions to evaluate treatment fidelity, instead we had one CC counselor attend most sessions. Fifth, this study did not collect placebo or sham control group data on sleep parameters. Due to this lack of data and the small sample size, our findings do not inform power analysis for future RCTs. Sixth, we did not plan the RCT using the RE-AIM framework. Thus, we did not establish a priori decision points to align the process with RE-AIM indicators in our clinical trial evaluation. Seventh, we are missing data on a portion of clients who attended the first screening session only. Next, at the time of this study, mOUD was inaccessible for HUM residents due to standard therapeutic community principles. The unabated rise in drug fatalities and urgent need for access to evidence-based SUD treatments has led to a paradigm shift in the role of mOUD in SUD recovery among therapeutic communities, including HUM. Future studies are warranted to further our understanding of the effectiveness of behavioral sleep interventions for individuals on mOUD. Finally, while we consider it advantageous to conduct clinical research among patients within a population the Health Resources and Services Administration defines as underserved [94], some may consider the present study population too complex and heterogeneous for analyses. Despite these limitations, we believe that this paper presents a transparent and pragmatic subjective evaluation of our clinical trial that offers novel insights into behavioral management of comorbid insomnia and SUDs and supports the design and implementation of future randomized trials that would benefit from recruitment across additional or larger settings.

## Conclusion

This is the first study to apply the RE-AIM framework to the evaluation of a RCT of an insomnia intervention conducted among a therapeutic community. These findings emphasize the importance of engaging multiple stakeholders in the adaptation of insomnia interventions among SUD treatment programs. Improved treatment of insomnia as part of comprehensive evidence-based outpatient SUD treatment programs may provide an efficient and cost-effective opportunity to improve care delivery. This study serves as a potential model for community-academic partnerships and therapeutic communities to apply implementation science in clinical research studies. It also suggests that further adaptation and evaluation of insomnia interventions may more efficiently shepherd clients towards individually tailored evidence-based interventions in hopes that these interventions promote recovery and longer treatment duration.

## Data Availability

The datasets used and/or analyzed during the current study are available from the corresponding author on reasonable request.

## Abbreviations

SUD: Substance use disorder
CBT-I: Cognitive behavioral therapy for insomnia
gCBT-I: Group-based cognitive behavioral therapy for insomnia
42CFR: Code of Federal Regulations 42, part 2
SOC: Standard of care
SOL: Sleep onset latency
SE: Sleep efficiency
WASO: Wake after sleep onset
TST: Total sleep time
HUM: Helping Up Mission
CC: Cornerstone Clinic
CARF: Commission on the Accreditation of Rehabilitation Facilities
RBT: Reinforcement-based treatment
ASAM: American Society of Addiction Medicine
IOP: Intensive outpatient treatment program
ISI: Insomnia Severity Index
PROs: Patient-reported outcomes
SIS-D: Structured Interview for Sleep Disorders
BAM: Brief addiction monitor
